# Endovascular treatment without postoperative decompressive craniectomy in an acute stroke patient with very large ischemic infarct core: A case report and literature review

**DOI:** 10.1016/j.heliyon.2024.e32172

**Published:** 2024-05-29

**Authors:** Chengchun Liu, Yulai Kang, Lili Zhang, Meng Zhang, Chunhua Tang

**Affiliations:** Department of Neurology, Daping Hospital, Army Medical University, Chongqing, 400042, China

**Keywords:** Endovascular treatment, Very large ischemic infarct core, Decompressive craniectomy, Functional independence, Case report

## Abstract

**Introduction:**

The benefits of endovascular treatment (EVT) on large ischemic infarct core mainly focus on a core size of 70–150 ml. The relationship between EVT and very large ischemic infarct core (>150 ml) is unclear. We herein present an acute stroke patient who achieved functional independence after EVT without postoperative decompressive craniectomy despite very large ischemic infarct core.

**Case report:**

A 50-year-old Asian male was admitted to our hospital with “sudden disturbance of consciousness with left limb weakness for 11 hours”. The patient had a history of clipping treatment for ruptured aneurysms. After an emergency CTA and CTP, very large ischemic core of 190 ml and a mismatch ratio (Tmax > 6s volume/core volume) of 1.9 were shown in preoperative imaging. EVT was performed, and postoperative strict monitoring was conducted without decompressive craniectomy. The patient was discharged from the hospital on the 16th day, scoring 2 on the modified Rankin scale at a 2-year follow-up.

**Conclusion:**

Imaging suggests very large ischemic infarct core; if there is a substantial mismatch between major functional areas (large ischemic penumbra) and the patient is relatively young, aggressive EVT may be beneficial.

## 1 Introduction

Ischemic stroke with large ischemic infarct core (70–150 ml) has the worst prognosis [1]; even after aggressive endovascular treatment (EVT), only 20 % of patients achieve functional independence (defined as a score on the modified Rankin scale [mRS] of 0–2) [2]. Six large clinical trials have confirmed the benefits of EVT for ischemic stroke patients with large ischemic infarct core [3]. However, it is still unclear whether very large ischemic infarct core (>150 ml) can benefit from EVT, and the current clinical evidence is insufficient [2]. If the ischemic infarct core was larger, the patients were less likely to achieve functional independence after EVT [4], the risk of intracranial hemorrhage is higher, and postoperative comprehensive care has become more stringent. We herein reported an acute stroke patient with very large ischemic infarct core (190ml), the patient's family provided the strongest consent when the family was fully informed of the benefits and risks of EVT and possible risks after postoperation. Subsequently, the patient underwent EVT without postoperative decompressive craniectomy and was discharged from the hospital on the 16th day. Regular visits to the outpatient clinic were made to assess the condition, and the patient achieved functional independence (mRS = 2) at a 2-year follow-up.

## 2 Case report

A 50-year-old Asian male was admitted to Stroke Green Pathway due to “sudden disturbance of consciousness with left limb weakness for 11 hours”. The patient was drowsy at admission, and the National Institutes of Health Stroke Scale (NIHSS) score was 13 (consciousness 1 + gaze 1 + facial paralysis 2 + left upper limb 4 + left lower limb 2 + sensory 1 + language 1 + articulation 1). The risk factors were found in this patient through inquiry. His parents both had high blood pressure (BP), and his father died of stroke; the patient himself smoked for at least 30 years, about 20 cigarettes per day; the homocysteine content was 33.16 μmol/L; the patient underwent right posterior communicating artery aneurysm clipping and ventriculostomy due to subarachnoid hemorrhage 2 years ago.

**Fig. 1:**
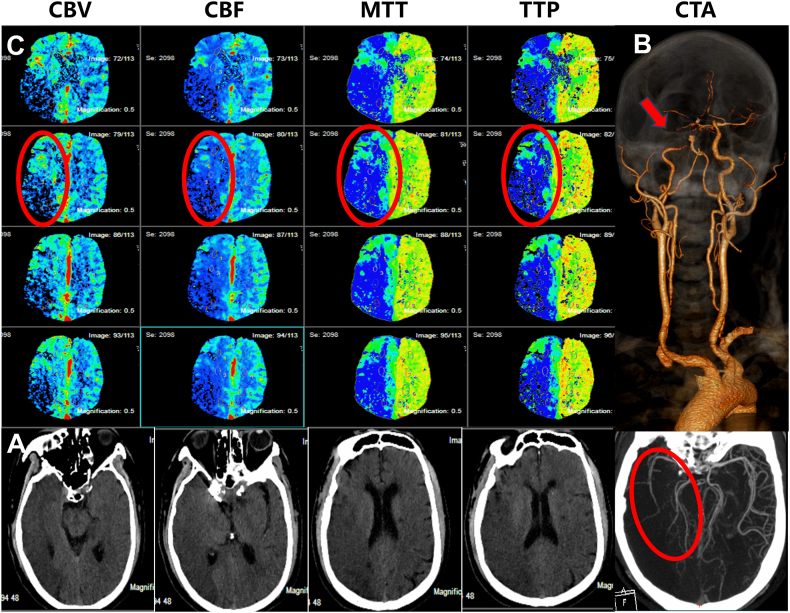
Preoperative one-stop CT examination. A: No hemorrhage was seen in the head CT scan. B: Head and neck CTA shows severe stenosis of the siphon segment of the right ICA (indicated by the yellow arrow) and sparse branching of the middle cerebral artery (indicated by the red arrow). C: Head CTP shows a large CBV area and CBF decline in the right cerebral hemisphere, extensive MTT and TTP prolongation, indicating the existence of a large-area cerebral infarction (shown in red circle). CBV cerebral blood volume; CBF-cerebral blood flow; MTT - mean transit time; TTP - time to peak. (For interpretation of the references to colour in this figure legend, the reader is referred to the Web version of this article.)

**Fig. 2:**
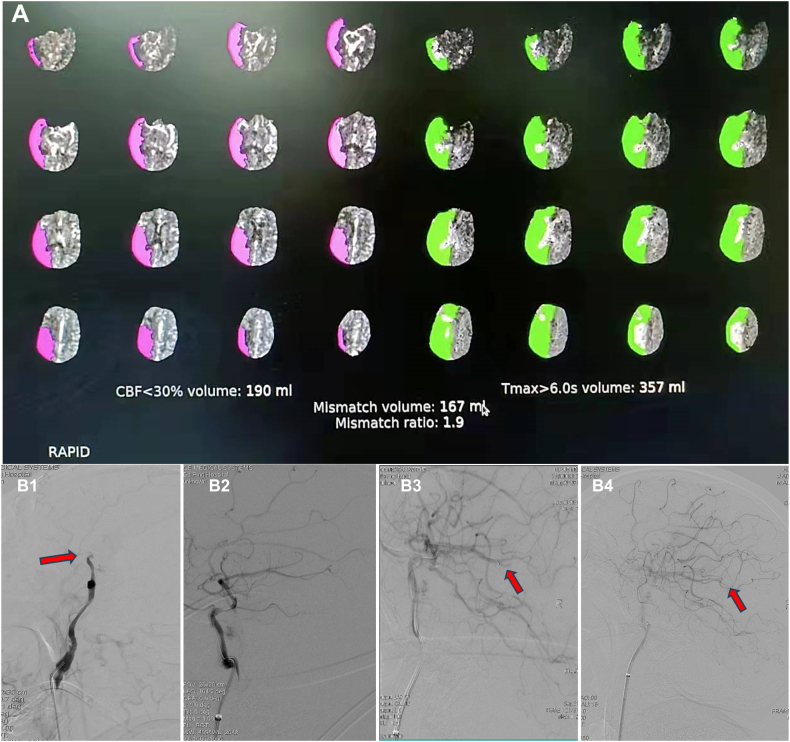
RAPID software estimated the core infarct volume, and endovascular intervention was performed. A: RAPID software estimated the great core infarct volume. B1: DSA showed occlusion of the C6 segment of the RICA (red arrow). B2: Positive microcatheter “first-pass effect”. B3: The Gateway (2.0 × 15mm) balloon was slowly pressurized, and a small branch of the right MCA-M3 was occluded. B4: Angiography showed approximately 70 % stenosis in the right ICA-C6, and right PCA, right ACA, and right MCA were well visualized (red arrow). (For interpretation of the references to colour in this figure legend, the reader is referred to the Web version of this article.)

An emergency head and neck computed tomography (CT) was done. The plain CT scan revealed postoperative changes in the intracranial aneurysms and large low-density lesions in the right cerebral hemisphere with an Alberta Stroke Program Early Computed Tomographic Score (ASPECTS) value of 2 (Fig. 1. A). CT angiography indicated low contrast signals at siphon and occlusion of the C6 segment in the right internal carotid artery (ICA), the right middle cerebral artery (MCA), and the right posterior cerebral artery (PCA) were sparsely branched, the anterior communicating artery was small, and the bilateral embryonic posterior cerebral arteries and basilar arteries (BA) had uneven thicknesses and segmental occlusions (Fig. 1. B). CT perfusion showed extensive hypoperfusion in the right cerebral hemisphere (Fig. 1. C), and RAPID software calculated that the ischemic infarct core volume (cerebral blood flow <30 %) was 190 ml, the mismatch volume (Tmax > 6s volume minus core volume) was 167 ml, and the mismatch ratio (Tmax > 6s volume/core volume) was 1.9 (Fig. 2. A).

The family agreed to EVT after careful consideration after fully communicating with the patient's family about his condition and the benefits and risks of EVT. Balloon dilation of the C6 segment of the right ICA was performed under local anesthesia. The EVT process was as follows: door to puncture was 30 minutes. After the successful puncture, 3000 U of heparin was injected intravenously, and 15 ml of tirofiban was injected intravenously, followed by a micro pump (10 ml/h). Angiography showed occlusion of the C6 segment (Fig. 2. B1), minor compensation of the intracranial blood supply by the right ophthalmic artery through small vessels at the base of the skull, partial compensation of the blood supply by the left anterior cerebral artery (ACA) through the anterior communicating artery to the right ACA and the right MCA, minor compensation of the blood supply to the posterior circulation by the left PCA through the P1 segment, and occlusion of the BA. A Synchro 0.014 in x 300 cm micro guidewire guided an Echelon-10 microcatheter through the occlusion segment to the right MCA-M1 segment (Fig. 2. B2). Microcatheterography confirmed that Echelon-10 was in the vascular lumen and that the micro guide wire was retained in the right MCA-M2 segment. Then, the microcatheter was removed; angiography showed right ICA-C6 segment stenosis greater than 90 %, slow forward blood flow, good visualization of the right ACA and right MCA, and intermittent intra-arterial bolus injection of tirofiban was performed. After 30 minutes of blood perfusion, a Gateway 2.0 × 15 mm balloon was used to slowly pressurize (6 atm/120 s) the balloon. Follow-up angiography showed approximately 80 % stenosis in the right ICA-C6 segment and improvement in the forward blood flow, and the right PCA, right ACA, and right MCA were well visualized. Seven minutes later, the Gateway 2.0 × 15 mm balloon was slowly pressurized (12 atm/120 s). The follow-up angiography showed approximately 70 % stenosis in the right ICA-C6 segment, and good forward blood flow and the right PCA, right ACA, and right MCA were well visualized, and a small branch of the right MCA-M3 was occluded (Fig. 2. B3); thrombolysis in cerebral infarction was grade 2C. Intermittent intra-arterial bolus injections of tirofiban and intermittent repeated angiography were performed. After 30 minutes of observation, the follow-up angiography showed approximately 70 % stenosis in the right ICA-C6 segment, good forward blood flow, and right PCA, right ACA, and right MCA were well visualized (Fig. 2. B4); the thrombolysis in cerebral infarction was grade 2C. During the operation, 1000U heparin was added, and 8 ml of tirofiban was injected intra-arterially.

**Fig. 3:**
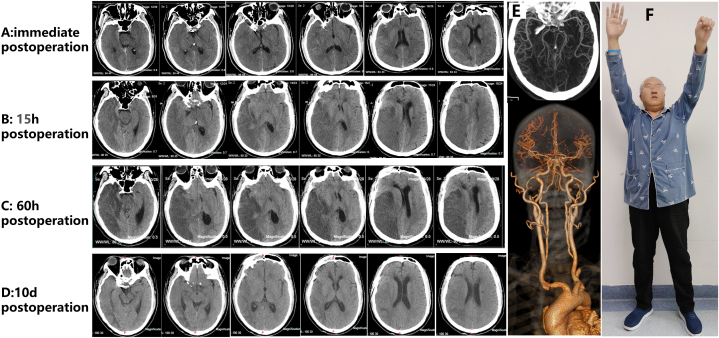
Postoperative follow-up. A: The head CT scan - immediate postoperation showed large hypodense foci in the right cerebral hemisphere with a midline shift. B. The head CT scan - 15 hours postoperation showed large low-density foci on the right side, and the brain tissue was swollen. C. The head CT scan - 60 hours postoperation showed large low-density foci on the right side. D. The head CT scan −10d postoperation showed the centered midline. E. Head and neck CTA -10d postoperation showed the siphon segment of the right ICA was stenotic (70 %). F. The patient's mRS score was grade 2 at the 2-year follow-up.

Dexmedetomidine sedation and sufentanil analgesia were continued after the operation. Immediate postoperative CT scan showed large low-density lesions in the right cerebral hemisphere with a midline shift (Fig. 3. A). Vital signs and level of consciousness were closely monitored, and the airway, blood pressure, body temperature, blood glucose, blood sodium, and nutrition were controlled in the neurological intensive care unit. The patient was pharmacologically sedated and received protective endotracheal intubation, ventilator-assisted respiration, continuous infusion of tirofiban (8 ml/h), heparin (300 U/h), strong dehydration to lower intracranial pressure (3 % hypertonic salt q6h, mannitol q6h, human serum albumin, and furosemide bid), and strict blood pressure control (110–120/70-80 mmHg). Two hours and 15 hours (Fig. 3. B) after the operation, CT showed low-density lesions on the right side, and the brain tissue was swollen. Transcranial Doppler (TCD) monitoring showed a high flow rate in the MCA on the affected side, indicating high intracranial pressure and a high risk of hemorrhagic transformation. The family was fully informed, and decompressive craniectomy was considered while closely monitoring the patient. Daily routine bedside TCD was conducted, changes in blood flow rate were observed, the blood pressure control range was adjusted based on test results, and bedside rehabilitation therapy was actively provided. The patient was switched to antiplatelet drug (aspirin, 100 mg/d; ticagrelor, 90 mg/d) treatment on the 4th day; the patient was conscious and underwent off-line training on the 5th day. On the 7th day, the patient was off-line, and on the 8th day, he was extubated. Head CT was performed at 32 hours, 60 hours (Fig. 3. C), 85 hours, 133 hours, 10th days (Fig. 3. D), and 16 days. Head and neck CTA was reexamined 10 days after the operation (Fig. 3. E), and the siphon segment of the right ICA was stenotic (70 %). The patient was discharged 16 days postoperatively. The NIHSS score was 7 points (facial paralysis 2+left upper limb 2+left lower limb 1+sensation 1+language 1). Magnetic Resonance Angiography showed right ICA patency with 50 % lumen stenosis, and the right MCA was well visualized. In the following 2 years, the patient continued to visit the outpatient department without interruption and insisted on taking antiplatelet drugs and statins, which effectively prevented the occurrence of ischemic stroke and ischemic events. A professional neurologist assessed the mRS score; the patient's mRS score was grade 2 at the 2-year follow-up (Fig. 3. F). Although the patient cannot continue working, he can handle his basic needs independently without assistance.

## 3 Discussion

Six randomized controlled trials (RESCUE-Japan LIMIT; ANGEL-ASPECT; SELECT-2; TESLA; LASTE; TENSION) of large ischemic infarct core (70–150 ml) revealed the effectiveness of EVT compared to the best medical treatment [[5], [6], [7], [8], [9], [10]]. However, whether patients with very large ischemic infarct core (>150 ml) continue to benefit from EVT is unclear [2]. Our case reported that a patient with very large ischemic infarct core benefited from EVT after professional evaluation by neurologists and achieved functional independence at a 2-year follow-up.

Although the six randomized controlled trials used slightly different methods to assess large ischemic infarct core, the benefits of EVT compared to the best medical treatment were clear and significant. RESCUE-Japan LIMIT study showed that EVT was found to be beneficial for patients with an ASPECT score of 4–5; however, the risk of intracranial hemorrhage was increased for patients with an ASPECT score ≤3, and only approximately 14 % of patients achieved functional independence [5,11]. TENSION study showed that about 17 % of patients with an ASPECT score of 3–5 achieved functional independence, and 9 % underwent decompressive craniectomy after EVT [6]. ANGEL-ASPECT study showed that about 30 % of patients with median infarct-core volume (60.5ml) achieved functional independence, and 49.1 % presented any hemorrhage after EVT [8]. SELECT-2 study showed that The benefit of EVT lasted up to 150ml of infarct-core volume, and 20.3 % of patients with median infarct-core volume (80ml) achieved functional independence [9]. LASTE study in the SLICE conference showed no difference in the therapeutic effect of EVT between patients with an ASPECT score of 0–2 and 3–5 [7]. TESLA study at the European Organization for Stroke Conference showed that the therapeutic effects of EVT were similar in patients with an ASPECT score of 2–3 and 4–5 [10]. Based on these, the National Clinical Guideline for stroke for the United Kingdom and Ireland recommends EVT for an ASPECT score ≥3 [12]; an ASPECT score of 0–2 is also likely beneficial after EVT. It is worth noting that although EVT has effectively improved mortality in patients with large ischemic infarct core, the risk of death in these patients is still as high as 35 %–40 %. In addition, the proportion of patients who underwent decompressive craniectomy after EVT was as high as 10 %, and the possibility of functional independence was only 20 %. High-quality data supporting EVT is lacking for patients with very large ischemic cores (>150 ml). How to carry out individualized treatment for such patients is also lacking [2].

In this case, preoperative imaging indicated very large ischemic infarct core of 190 ml and an ASPECT score of 2. The decision to perform EVT was made thanks to the families' strongest support and the doctors’ dedication to saving lives. Now, it seems very true indeed. This patient achieved functional independence, and possible explanations include: (1) The patient still had residual ASPECT score and low NIHSS score despite very large ischemic core, which may be attributed to the relative youth of the patient and the fact that severe cerebrovascular stenosis has been present for a long time before onset. Incomplete cytotoxic edema occurs in many neurons due to ischemic preconditioning and collateral circulation [11], which may achieve functional recovery with blood flow recovery. However, for patients with embolic acute ischemic stroke, clinical outcomes may be worse due to a lack of adequate ischemic preconditioning and collateral circulation compensation [12]. (2) The choice of simple balloon dilatation quickly realized vascular recanalization and greatly shortened the intervention time [13]. Residual stenosis prevents hyperperfusion injury. (3) Perioperative management is also critical. The patient was postoperatively sent to an independent neurointensive care unit for management. Postoperative deep sedation and analgesia, strict blood pressure control, intracranial pressure lowering with multiple osmotic drugs, and strict bedside TCD monitoring helped the patient pass the edema period safely; decompressive craniectomy was avoided. (4) During follow-up, the patient was able to continue taking antiplatelet drugs and statins [14], which prevented the recurrence of stroke and ischemic events. The patient achieved functional independence at a 2-year follow-up.

Limitations exist in this case report. Firstly, the time from onset of the disease to arrival at the hospital was 11 hours, which to some extent affected the further recovery of the patient's clinical prognosis. Secondly, although regular outpatient follow-up was conducted, functional outcomes were only assessed in the second year of the disease. Furthermore, it is unclear how the patient's post-EVT recovery treatment should proceed, as rehabilitation therapy can still improve the patient's clinical prognosis to some extent.

## 4 Conclusion

In short, aggressive EVT may benefit young patients with acute stroke, even those with very large ischemic core, if imaging suggests a large mismatch in major functional areas (larger ischemic penumbra). In our experience, multimodal imaging evaluations should be conducted before EVT to clarify brain histological information; postoperatively, patients should be strictly managed in an independent neurointensive care unit. With advancements in surgical equipment and materials, the optimization of workflows, the development of precise imaging technology, and the introduction of artificial intelligence, an increasing number of patients with very large ischemic core may benefit from EVT.

## Ethics statement

The study was approved by the Ethics Committee of Daping Hospital.

## Consent for publication

Written informed consent was obtained from patient.

## Funding

This study was supported by 10.13039/501100005230Natural Science Foundation of Chongqing. (CSTB2022NSCQ-MSX1585).

## Data availability statement

The data that support the findings of this study are available from the corresponding author upon reasonable request.

## CRediT authorship contribution statement

**Chengchun Liu:** Writing – review & editing, Writing – original draft, Investigation, Data curation. **Yulai Kang:** Writing – review & editing, Writing – original draft, Investigation, Data curation. **Lili Zhang:** Visualization, Supervision, Formal analysis. **Meng Zhang:** Writing – review & editing, Writing – original draft. **Chunhua Tang:** Writing – review & editing, Writing – original draft.

## Declaration of competing interest

The authors declare that they have no known competing financial interests or personal relationships that could have appeared to influence the work reported in this paper.
